# Identification of triple gene fusion ALK-LRRN2, LTBP1-ALK, and HIP1-ALK in advanced lung adenocarcinoma and response to alectinib: A case report

**DOI:** 10.1097/MD.0000000000027999

**Published:** 2021-12-23

**Authors:** Shangkun Ning, Congcong Shi, Huifang Zhang, Jinpeng Li

**Affiliations:** a Shandong Cancer Hospital and Institute, Shandong First Medical University and Shandong Academy of Medical Sciences, Jinan, Shandong, 250117, P.R. China; b Shandong Mental Health Center, Jinan, Shandong, 250114, People's Republic of China; c Interventional Therapy Department Ward 1, Shandong Cancer Hospital and Institute, Shandong First Medical University and Shandong Academy of Medical Sciences, Jinan, Shandong, 250117, P.R. China.

**Keywords:** alectinib, ALK-LRRN2, HIP1-ALK, LTBP1-ALK, lung adenocarcinoma, triple fusions

## Abstract

**Rationale:**

Anaplastic lymphoma kinase (ALK) rearrangement is the second most common targetable oncogene-dirven gene in nonsmall cell lung cancer. Owing to the advanced sequencing technologies, new partner genes of ALK have been constantly detected.

**Patient concerns:**

A 42-year-old Chinese woman went to our hospital with the chief complaint of cough and expectoration for 1 month. The patient had no fever, chest pain, and hemoptysis.

**Diagnoses:**

She was diagnosed with advanced lung adenocarcinoma. The patient underwent lung biopsy guided by computed tomography and pathology showed poorly differentiated adenocarcinoma. To explore possibility of targeted therapy, the tumor samples were subjected to next-generation sequencing, and a rare 3 ALK fusion variant ALK-LRRN2, LTBP1-ALK, and HIP1-ALK was identified.

**Interventions and outcomes:**

The patient subsequently received alectinib treatment, and achieved partial response. No significant drug related adverse reactions were found during alectinib treatment. The progression-free survival achieved 25 months.

**Lessons:**

Together, we identified a rare triple ALK fusion variant, ALK-LRRN2, LTBP1-ALK and HIP1-ALK, in a patient with lung adenocarcinoma. The patient benefited from alectinib treatment, which could provide a certain reference for the patients with such gene alteration.

## 1 Introduction

About 30% to 50% of patients with advanced nonsmall cell lung cancer (NSCLC) will have brain metastasis, and the natural course of these patients is short, which was just 3 months. Anaplastic lymphoma kinase (ALK) rearrangement is the second most common sensitive driver gene in NSCLC. It is identified in approximately 3% to 7% of NSCLC patients,^[1,2]^ and is highly sensitive to ALK tyrosine kinase inhibitors (TKIs) such as crizotinib, alectinib, ceritinib, brigatinib.^[3,4]^ In the era of cancer precision therapy, with the continuous optimization and update of gene sequencing technology, more and more atypical ALK fusion genes have been detected. EML4-ALK rearrangement was firstly identified by Togashi et al in 2012.^[5]^ With the development of next-generation sequencing (NGS), more and more novel ALK fusion partners have been identified, such as KIF5B, STRN, KLC1, TPR, HIP1, DCTN1, LTBP1,GCC2, SEC31A.^[2]^ Herein, we report a rare fusion of 3 genes leucine rich repeat neuronal 2(LRRN2), latent transforming growth factor β binding protein 1(LTBP1) and huntingtin interacting protein 1 (HIP1)-ALK fusion in a patient with NSCLC using NGS.

## 2 Case report

**Figure 1:**
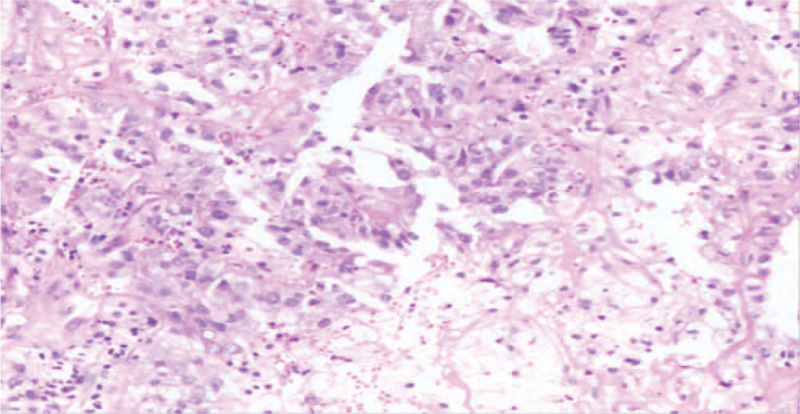
Histological findings from biopsy specimens: poorly differentiated adenocarcinoma (×100) (H&E).

**Figure 2:**
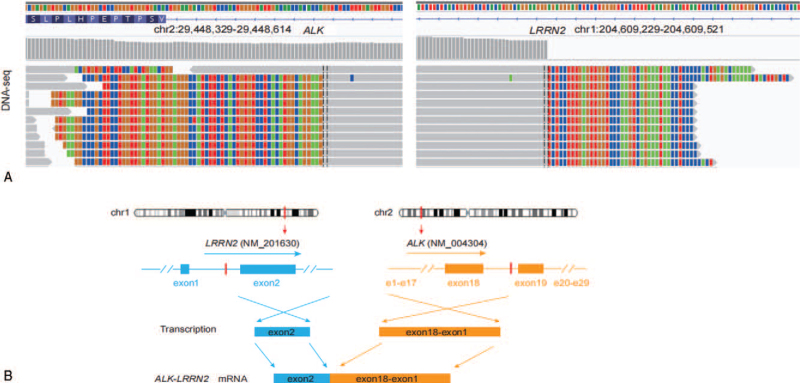
Concomitant LRRN2-ALK fusion was found. (A) Demonstrated the DNA sequencing reads indicating fusion region by genomics Viewer (IGV) software. (B) Showed the breakpoints detected in the fusion by targeted NGS and the alterations in gene caused by this fusion.

**Figure 3:**
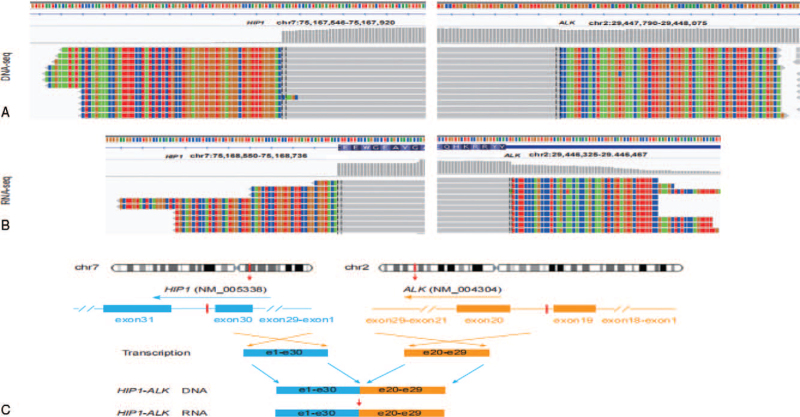
Concomitant HIP1-ALK fusion was found. (A) Demonstrated the DNA sequencing reads indicating fusion region by genomics Viewer (IGV) software. (B) RNA sequencing reads indicating this fusion region demonstrated by IGV. (C) Showed the breakpoints detected in the fusion by targeted NGS and the alterations in gene, mRNA and protein caused by this fusion.

**Figure 4:**
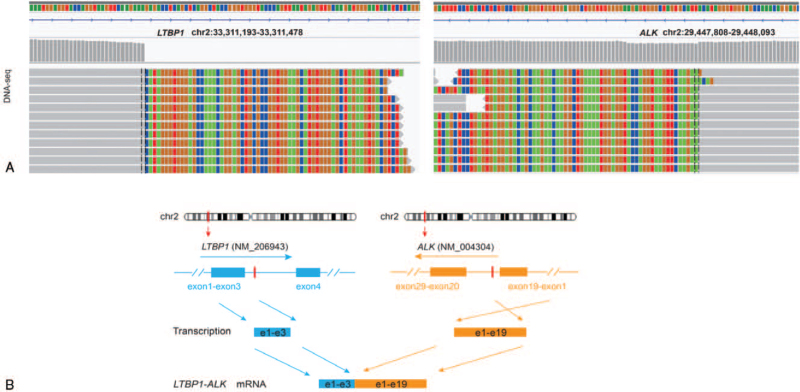
Concomitant LTBP1-ALK fusion was found. (A) Demonstrated the DNA sequencing reads indicating fusion region by genomics Viewer (IGV) software. (B) Showed the breakpoints detected in the fusion by targeted NGS and the alterations in gene caused by this fusion.

**Figure 5:**
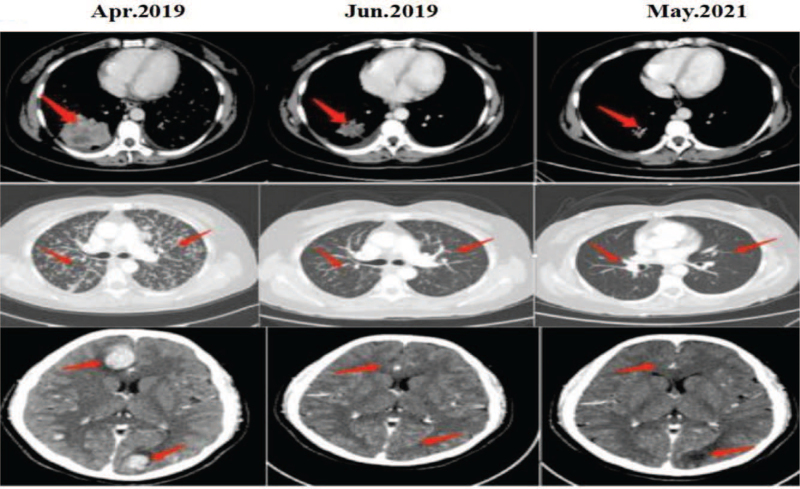
Radiographic response to treatment. April 2019: chest CT scan performed before alectinib treatment (March 10, 2020) showed a 6.7 × 5.4 cm irregular mass and multiple enlarged lymph nodes can be seen in both hilum, mediastinum and double clavicle, and the large one have a short diameter of about 1.2 cm; Multiple enhanced nodules and masses can be seen in bilateral brain parenchyma, with a diameter of about 2.7 cm and a low density edema zone around part of the brain. June 2019: CT performed after 45 days of alectinib treatment (April 27, 2019) demonstrated a reduced tumor volume (2.7 × 3.4 cm) and the nodules and masses in the bilateral brain parenchyma were not clearly displayed, and there were low-density edema areas around the brain. All were dramatically reduce compared with the previous. May 2021: the intrapulmonary lesion was significantly smaller than before; lamellar hypodensity with indistinct borders was seen in the right frontal and left occipital lobes. Efficacy evaluation: PR.

A 42-year-old Chinese woman was admitted to our hospital with the chief complaint of cough and expectoration for 1 month. The patient was previously fit and had no history of familial tumor-related disease. Computed tomography (CT) (April 16, 2019) scan showed an irregular mass measuring 6.7 cm plus 5.4 cm and multiple enlarged lymph nodes can be seen in both hilum, mediastinum and double clavicle. Multiple enhanced nodules and masses can be seen in bilateral brain parenchyma, with a diameter of about 2.7 cm and a low density edema zone around part of the brain (Fig. 5). The patient underwent lung biopsy guided by CT and pathology showed poorly differentiated adenocarcinoma (Fig. 1). After comprehensive evaluation, the patient was diagnosed as lung adenocarcinoma (cT3N2M1c, stage IVB). Specific immunohistochemical results were as follows: TTF1(+), Napsin A(+), CK7 (+), P63(−) and CK5/6(−). NGS was performed and 3 rare fusion genes LRRN2-ALK (abundance: 2.74%), LTBP1-ALK (abundance: 10.28%), and HIP1-ALK (abundance: 9.38%) were identified (Figs. 2–4). Other genes like EGFR, KRAS, and ROS1 were negative.

The patient was treated with alectinib with the dose of 600 mg twice a day. One month later, the patient's symptoms were improved and the primary and metastatic tumors were reduced significantly (Fig. 5). Partial response was achieved according to RECIST 1.1. The patients were treated with alectinib thereafter, with a follow-up of 25 months and the response of partial response was maintained until May 2021. Patients presented with a mild rash and no treatment associated adverse events were found according to CTCAE grades.

Written informed consent has been provided by the patient to have the case details and any accompanying images published.

## 3 Discussion

At present, the main ALK fusion gene found in lung cancer is EML4 fusion ALK, which accounts for about 4% and 7%, which has been proved to be an independent and key molecular target in the occurrence and development of NSCLC.^[6,7]^ With the development of gene sequencing technology, more and more atypical ALK fusion genes have been detected. Compared with NSCLC without rearrangement, the NSCLC stage of ALK gene rearrangement is later, and the brain is the most common organ of tumor metastasis and progression.^[8,9]^ A retrospective study showed that the cumulative incidence of brain metastasis in 2 years and 3 years in patients with ALK-NSCLC was 45.5% and 58.4%, respectively.^[10]^

In recent years, with the continuous development and clinical application of ALK- TKI, the progression-free survival (PFS) and overall survival (OS) of intracranial diseases with ALK-NSCLC brain metastasis have been significantly prolonged,^[11]^ but how to use each generation of ALK-TKI reasonably and effectively to treat patients with NSCLC brain metastasis is worth exploring. Crizotinib is a small molecular multi-target TKI, which can inhibit ALK, c-MET, and ROS-1 fusion proteins. It has been approved for first-line treatment of ALK-NSCLC patients in the United States, China, Japan, Europe, and other countries and regions. Crizotinib was the first clinical ALK inhibitor for late ALK positive patients. In patients with NSCLC, the drug was significantly better than chemotherapy, but the clinical effect is not good in patients with baseline brain metastasis.^[12]^ A retrospective study^[13]^ showed that 72% of 60 patients with NSCLC brain metastases who received local intracranial treatment followed by crizotinib had a second progression of central nervous system (CNS). Of the NSCLC patients without brain metastasis at baseline, 20% developed brain metastasis during the treatment of crizotinib, which may be related to the low permeability of blood–brain barrier. CNS metastasis is the main factor for the failure of clozotinib treatment. Alectinib was a kind of ALK-TKIs with high CNS permeability and high selectivity. It could inhibit most of the acquired crizotinib resistance mutations, including G1269A, L1196 M, C1156Y, L1152R, F1174L, 1151Tins, and so on. Nakagawa et al^[14]^ reported that alectinib treated NSCLC 207 patients resistant to crizotinib with a median PFS of 8.1 months (95% confidence interval: 6.2∼12.6) and a 12-month OS of 71% (95% confidence interval: 61∼81). Based on these 2 studies, the alectinib was approved for the treatment of ALK-NSCLC patients who had progressed after treatment with crizotinib by FDA in December 2015. Patients with ALK-NSCLC brain metastasis could also benefit from alectinib treatment. The AF-001JP trial by Seto et al^[15]^ reported that the 3-year PFS of alectinib (600 mg, bid) in 70 patients with ALK-NSCLC was 62%, and the 3-year OS was 78%. The median PFS and OS had not been reported, and 6 of the 14 patients with brain metastasis had no intracranial tumor progression within 3 years. For patients with NSCLC brain metastasis with definite ALK gene fusion, alectinib was recommended to consider both CNS and systemic efficacy and tolerance.^[16,17]^

ALK fusion chaperone-mediated ALK fusion does not depend on its ligand to form dimer, which activates the protease domain and leads to tumorigenesis. Studies have shown that ALK fusion gene positive tumor anaplastic large cell lymphoma, whose fusion companion is nucleolar phosphoprotein, is considered to be the main cause of increased ALK activity and ALCL.^[18]^ The mechanism remains to be explored. But, the first fusion ALK-LRRN2 is at 5’ and the chaperone gene is at 3’ end. If there was only 1 gene fusion, the patient would not be able to use crizotinib drugs. But this patient also had 2 ALK fusion ALK at the 3’ end, so this fusion gene contains the ALK kinase domain sequence, which could translate the kinase domain protein, and could use ALK inhibitors, although fusion may be rare or new, as long as the ALK20-28 exon was retained, it would not affect the use of drugs.

In this case report, a new rare fusion form of ALK rearrangement (ALK-LRRN2, LTBP1-ALK, and HIP1-ALK) was identified using the NGS, based on the DNA sequencing data. The LRRN2 gene is located on human chromosome 1, and its protein product is rich in light. The amino acid repeat unit superfamily is a transmembrane protein, which may be related to cell adhesion and signal transduction. Studies have shown that it is abnormally expressed in a variety of brain tumors, such as malignant gliomas and the abnormal amplification rate of LRRN2 gene is close to 50%.^[19]^ LTBP1 is located on the chromosomal band 2p22.3, and take part in the assembly and secretion of the latent TGFβ1, HIP1 is over-expressed in many human cancer cell lines, which suggests that HIP1 can provide selective growth advantage for cancer cells. LTBP1 and HIP1 ALK fusion is ALK at the 3’ end, then the fusion gene contains ALK kinase domain sequence, which can translate the kinase domain protein, thus promoting the occurrence of lung tumors, so ALK inhibitors can be used. According to study guidelines, crizotinib is recommended as a first-line treatment for locally advanced or metastatic ALK positive NSCLC.^[20]^ Although many fusion partners of ALK have been found in lung cancer, not all fusion mutations respond to crizotinib, such as CMTR1-ALK, which was reported has no response to crizotinib. The mechanism remains to be explored. Studies have shown that alectinib can significantly prolong the median survival time of patients with mild side effects, and the patient finally decided to use alectinib targeted therapy. The patient is still being treated with alectinib 600 mg PO BID.

In summary, we identified a rare 3-gene targeted ALK fusion by using powerful NGS-based tissue. The patient's positive response to alectinib has provided a better understanding of ALK-TKI applications in the future. Rare ALK fusion genes have a far-reaching impact on the efficacy of ALK-positive TKI.^[21]^ In patients with advanced ALK-positive NSCLC, some rare fusion genes may produce primary resistance to crizotinib, while others may be more effective than EML4-ALK fusion in ALK-TKI therapy.

## Author contributions

**Conceptualization:** Huifang Zhang, Jinpeng Li.

**Formal analysis:** Huifang Zhang, Congcong Shi.

**Methodology:** Jinpeng Li.

**Resources:** Congcong Shi, Jinpeng Li.

**Supervision:** Jinpeng Li.

**Validation:** Jinpeng Li.

**Writing – original draft:** Shangkun Ning, Congcong Shi.

**Writing – review & editing:** Huifang Zhang, Shangkun Ning, Congcong Shi.
